# Associations of early adulthood life transitions with changes in fast food intake: a latent trajectory analysis

**DOI:** 10.1186/s12966-020-01024-4

**Published:** 2020-10-09

**Authors:** Eleanor M. Winpenny, Megan R. Winkler, Jan Stochl, Esther M. F. van Sluijs, Nicole Larson, Dianne Neumark-Sztainer

**Affiliations:** 1 MRC Epidemiology Unit, University of Cambridge, Cambridge, UK; 2 Division of Epidemiology and Community Health, School of Public Health, University of Minnesota, Minneapolis, USA; 3 Department of Psychiatry, University of Cambridge, Cambridge, UK; 4 Department of Kinanthropology, Charles University in Prague, Prague, Czech Republic

**Keywords:** Fast food, Young adult, Life transition, Diet, Education, Employment, Partner, Parenthood, Longitudinal

## Abstract

**Background:**

Early adulthood is a period of rapid personal development when individuals experience major life transitions (e.g. leaving the parental home, leaving education, beginning employment, cohabitation and parenthood). Changes in social and physical environments associated with these transitions may influence development of health-related behaviours. Consumption of fast food is one behaviour associated with poor diet and long-term health outcomes. In this study we assess how frequency of fast food consumption changes across early adulthood, and how major life transitions are associated with changes in fast food intake.

**Methods:**

Data were collected across four waves of the Project EAT study, from mean age 14.9 (SD = 1.6) to mean age 31.1 (SD = 1.6) years. Participants reporting data at two or more waves were included (*n* = 2902). Participants reported past week frequency of eating food from a fast food restaurant and responded to questions on living arrangements, education and employment participation, and having children. To assess changes in fast food we developed a latent growth model incorporating an underlying trajectory of fast food intake, five life transitions, and time-invariant covariates.

**Results:**

Mean fast food intake followed an underlying quadratic trajectory, increasing through adolescence to a maximum of 1.88 (SE 0.94) times/week and then decreasing again through early adulthood to 0.76 (SE 2.06) times/week at wave 4. Beginning full-time employment and becoming a parent both contributed to increases in fast food intake, each resulting in an average increase in weekly fast food intake of 0.16 (*p* < 0.01) times/week. Analysis of changes between pairs of waves revealed stronger associations for these two transitions between waves 1–2 (mean age 14.9–19.4 years) than seen in later waves. Leaving the parental home and beginning cohabitation were associated with decreases in fast food intake of − 0.17 (*p* = 0.004) and − 0.16 (*p* = 0.007) times/week respectively, while leaving full-time education was not associated with any change.

**Conclusions:**

The transitions of beginning full-time employment and becoming a parent were associated with increases in fast food intake. Public health policy or interventions designed to reduce fast food intake in young adults may benefit from particular focus on populations experiencing these transitions, to ameliorate their impact.

## Background

Early adulthood (age 18–30 years) is the life course stage when prevalence of overweight and obesity increases the fastest [1]. The poor quality diet often consumed at this age [2] is likely to contribute to this increase. Behaviours established during early adulthood, including obesity-related behaviours such as dietary intake, eating behaviours and physical activity behaviours, may persist into later adulthood [3], influencing the risk of non-communicable disease in later life [4, 5]. Early adulthood has been identified as an important age for health behaviour interventions, which may be more successful at a time when habits are disrupted by lifestyle changes [6]. There is a need to build a better understanding of factors affecting changes in diet during early adulthood, and how this contributes to the establishment of long-term dietary behaviours to inform the development and targeting of diet interventions.

One negative aspect of people’s dietary intake that has increased in recent decades is consumption of food from fast food restaurants [7, 8]. In the U.S., the highest consumption of fast food is reported by young adults (aged 20–39); 45% of this population subgroup reports consuming fast food on any given day [9]. In the UK, young adults (age 16-24 yrs) were also the age group reporting highest fast food consumption, with 54% reporting eating in a fast food restaurant in the past month [10]. Frequent fast food consumption is associated with lower overall diet quality [2, 11], and longitudinal research has shown that higher frequency of fast food consumption in young adulthood is prospectively associated with increases in body weight and insulin resistance over the following 15 years [12].

Few studies have investigated longitudinal changes in fast food intake with age. Analysis of data from The National Longitudinal Study of Adolescent Health (Add Health), showed an increase in fast food consumption from age 16 (2.15 (SE 0.05) times per week) to age 21 (2.48 (SE 0.05) times per week) [13]. Similarly, previous analysis of the Project EAT (Eating and Activity in Teens and Young Adults) cohort has reported an initial increase in prevalence of high fast food intake (3 or more times per week) from adolescence (mean age 14.8 y (SD 1.6)) to early adulthood (mean age 19.4 y) with a maximum prevalence of 27.8% of males and 25.8% of females consuming fast food three or more times per week at this age, followed by subsequent decreases across time points in early adulthood (mean age 25.3 y and 31.0 y) [14]. Little is known regarding what social and environmental factors might contribute to changes in fast food intake over this period [15].

Early adulthood is a period of rapid personal development [16], as well as a time when individuals experience major changes in their social and physical environments and financial resources, identified as determinants of health behaviour [17]. In particular, there are five major life transitions which are likely to occur in early adulthood: moving out of the parental home, leaving education, beginning employment, cohabitation with a significant other, and becoming a parent [18, 19]. Some of these transitions have previously been found to be associated with changes in adiposity and physical activity. For example, transitions from high school to college, and the transition to becoming a parent have been found to be associated with increases in body weight, and leaving high school is associated with decreases in physical activity [20, 21]. However, there is little evidence of these transitions leading to changes in eating behaviours or dietary intake. A study of Norwegian data reports decreases in fruit and vegetable intake on leaving the parental home and increases in confectionery and sugar-sweetened beverage intakes on leaving education [18]. A small number of studies suggest that consumption of fruit, vegetables and dairy may decrease across the transition from high school to college [21], but report little change in dietary intake in those becoming parents [20]. Building a better understanding of the processes and mechanisms that shape diet and eating behaviours during early adulthood will be crucial to inform how best to intervene to promote the establishment of healthy behaviours and a high quality diet that persists from early to later adulthood.

In this study we aimed to address the research question: “How are the life transitions of early adulthood associated with changes in fast food intake?”. To address this question we: (1) described the prevalence and timing of the five life transitions in our population of US adolescents and young adults, (2) modelled the underlying trajectory of change in fast food intake with age across early adulthood, and (3) modelled associations between each life transition and change in fast food intake, accounting for the underlying trajectory, other life transitions, and time-invariant covariates.

## Methods

### Participants

Data for the current analysis were collected in Project EAT (Eating and Activity in Teens and Young Adults), a longitudinal study of weight-related health. In 1998–1999 (wave 1), a cross-sectional investigation of students at 31 public secondary schools in the metropolitan area of Minneapolis-St. Paul, Minnesota, U.S. was conducted. Public middle schools and high schools in the Minneapolis/St. Paul metropolitan area, serving socioeconomically and racially/ethnically diverse communities, were invited to participate in the study. Of 53 schools contacted, 31 agreed to take part. Participants aged 11 to 18 (mean age 14.9, *n* = 4746) completed surveys during class time, and height and weight measurements were taken by trained research staff in a private area [22, 23].

Given growing interest in weight-related health, a decision was made to follow-up with participants who provided sufficient contact information (*n* = 3672) at 5-year intervals. Follow-up assessments were completed by mail and online in 2003–04 (wave 2, *n* = 2516), 2008–09 (wave 3, *n* = 2287) and 2015–16 (wave 4, *n* = 1830) as participants progressed through adolescence and early adulthood [24]. At each wave participants completed a survey of around 100 questions, focused on diet, eating behaviours and other weight-related behaviours. At the final follow-up, wave 4, invitations to complete the survey were mailed along with a two-dollar bill to all participants who had responded to at least one previous follow-up survey. Survey invitation letters provided the web address and a unique password for completing the online version of the survey. Non-responders received up to six reminders through a combination of U.S. mail, email, and text messages. The final two mailed reminders included paper copies of the survey and FFQ, and all mailings provided the option to complete the survey by phone. Reminder emails were additionally mailed to participants that did not complete the survey and FFQ after logging into the online version. Participants were mailed a financial incentive ($50) following survey completion. The University of Minnesota’s Institutional Review Board Human Subjects Committee approved all protocols, with parental consent and participant assent obtained at wave 1 and participant consent obtained at each of the subsequent waves. In this study, given the focus on life transitions between waves of data collection, individuals were included in the analysis if they reported data at 2 or more waves of Project EAT (*n* = 2902), and therefore provided data that covered at least one period during which a transition may occur.

### Exposures

To allow for longitudinal comparisons, each wave of the EAT study included identical measures of key constructs. We report the psychometric properties of measures using data collected for Wave 4. The estimates of item test-retest reliability, reported below, were determined in a subgroup of 103 participants who completed the Wave 4 survey twice within a period of one to 4 weeks. The EAT surveys included measures of each of the five major life transitions that was based on assessment of living arrangements, participation in education and employment, and having children. Below we describe the measures used to assess each transition and how these were used to create the exposure variable used in the analysis. In a small number of cases (less than 10% of the population for each exposure) individuals who had gone through a life transition returned to pre-transition state (e.g. they moved back into the parental home). As we were primarily interested in the first occurrence of a life transition, we coded any data points after the return to the pre-transition state as missing, thereby removing them from the analysis. For example, if a participant reported living with parents at wave 1, not living with parents at wave 2, but then reported living with parents again at wave 3, the variable “leaving the parental home” would be coded as missing at wave 3 and wave 4 for that participant. This only applied to a small number of participants and did not contribute greatly to missing data.

*Leaving the parental home* was measured by asking participants about their current living arrangements at Waves 2–4. Participants were asked to select all that applied of nine options (e.g., “my parent(s)”) in answering “During the past year, with whom did you live the majority of the time?”. As this item was not asked when participants were under age 18, we assumed all participants were living with their parent(s) at Wave 1 and at Wave 2 if they were still a high school student. Based on the selection of “my parents” (Test-retest agreement = 97%) from the list of provided response options, participants were categorized as ‘living with parents’ or ‘not living with parents’ at waves 2–4. The transition variable of ‘leaving the parental home’ was positive the first time participants reported not living with parents.

*Leaving full-time education* was based on data about current educational status collected at waves 2, 3 and 4 in response to the question: “Which of the following best describes your student status (for the majority of the past year)?” (Test-retest agreement = 95%). Responses included type of education (e.g. community or technical college, four-year college) and whether education was full or part-time. Since Wave 1 surveys were administered in school classrooms, we assumed that all participants were attending full-time education at this time point. Graduate studies (reported at waves 3 and 4) was included under part-time education since no information was available as to whether graduate study was full-time or part-time, and data from the U.S. Department of Education suggests that 64% of graduate students completed their studies as part-time students in 2015–16 [25]. Less than 8% of the sample reported graduate studies at Waves 3 and 4, so this assumption is not likely to strongly affect our results. The transition variable of ‘leaving full-time education’ was positive the first time that participants reported participation in part-time or no education.

*Beginning full-time employment* was based on data about current employment status, derived in response to the question: “How many hours a week do you work for pay?” (Test-retest r = 0.80). Response options differed for those still in high-school compared to subsequent questionnaires. Those still in high-school only reported hours of work up to a maximum of ‘20 or more hours a week’; we categorised these individuals as participating in part-time work since they were still in full-time education. For those who had completed high-school, we categorised full time work as 30 or more hours per week [26]. The transition variable of ‘beginning full-time employment’ was positive the first wave that participants reported full-time work.

*Beginning cohabitation with a significant other* was based on responses to the current living situation measure that is described above. Participants who selected “my husband/wife” (Test-retest agreement = 93%), “my partner of the opposite sex” (Test-retest agreement = 92%), or “my partner of the same sex” (Test-retest agreement = 100%) were categorized as cohabiting with a significant other. As above, since this question item was not asked when participants were under age 18, we assumed all individuals at wave 1 and those who were still high school students at wave 2 did not live with a partner. The transition variable of ‘beginning cohabitation’ was positive the first time that participants began to report living with a partner.

*Becoming a parent* was based on responses to survey questions that asked participants to report how many children they had at waves 2, 3, and 4 (Test-retest r = 0.98). This question was not asked at wave 1, and it was assumed that participants did not have any children at wave 1, unless further detailed data on child age collected at wave 4 indicated that they would have already had a biological child at wave 1 (*n* = 10). 'Becoming a parent' was positive the first time that individuals moved from having no children to having one or more child at a subsequent wave.

### Outcomes

Fast food consumption frequency was reported at each wave in response to the question: “In the past week, how often did you eat something from a fast food restaurant (like McDonald’s, Burger King, etc.)?”. Response categories were converted to a continuous scale of times per week: never [recoded to 0 times per week], 1–2 times [1.5], 3–4 times [3.5], 5–6 times [5.5], 7 times [7], more than 7 times [10], as done previously [27]. This measure was developed for the Project EAT study and is comparable to established measures used by other studies such as the National Longitudinal Study of Adolescent Health [13].

### Covariates

We included baseline sociodemographic variables that may be associated with both life transitions and underlying changes in fast food intake as time-invariant covariates. These include age, gender, race/ethnicity, parental socio-economic status (SES) and health status [9, 15]. Baseline age of the participant in years and months, and gender were reported at wave 1. Race/ethnicity was also reported at wave 1 and collapsed to give two categories: (1) White (non-Hispanic) and (2) People of Colour. Parental SES was primarily determined from parental education, defined by the highest level of education achieved by either parent, as reported by the participants at Wave 1. This measure was complimented by additional variables on family eligibility for public assistance (yes, no, and do not know), eligibility for free or reduced-cost school meals (yes, no, and do not know), and maternal and paternal employment status (full-time, part-time, not working, and do not know), as described previously [28], to give a 5-level variable from 1 (low SES) to 5 (high SES). Overall health status was reported at waves 1 and 2 in response to the question “How would you describe your health?”, with response options poor (1), fair (2), good (3) or excellent (4). In order to provide a longer-term measure of health rather than only a snapshot at the time the initial questionnaire was completed, responses were averaged across waves 1 and 2 to represent baseline health status.

### Statistical analysis

Descriptive analyses were performed to evaluate sample population demographics and changes in fast food intake and occurrence of life transitions across the 4 waves. We tested for differences between the sample demographics of those included and excluded from our analysis (age, gender, race/ethnicity, parental SES and baseline health status) using linear regression and chi-squared tests. We calculated the phi coefficients of associations between pairs of transitions, to assess the likely influence of collinearity.

**Fig. 1:**
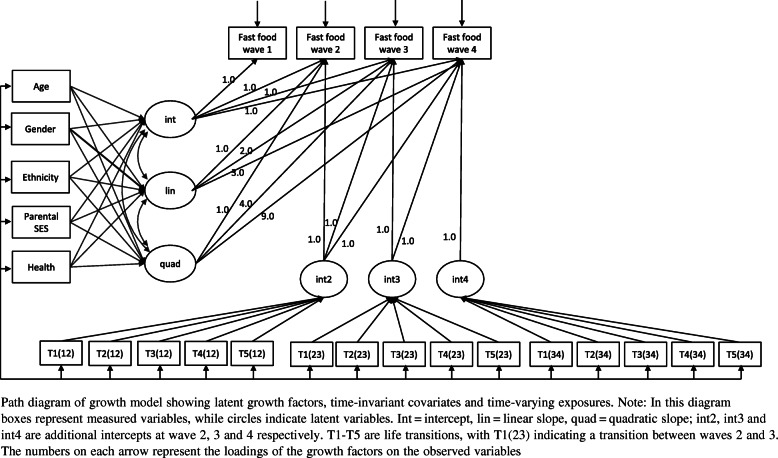
Path diagram of growth model showing latent growth factors, time-invariant covariates and time-varying exposures

Latent growth curve models were used to test our research questions. Growth models were developed as a hierarchy of increasing complexity. First an unconditional growth model was estimated to examine overall growth trajectories and to test for individual variability in change over time. We tested the main effects of 5 time-invariant covariates: baseline age, gender, ethnicity, parental SES and baseline health status, including these in the model as predictors of the intercept, slope and quadratic slope of the growth curve (Fig. 1). Additional latent factors were then included to test for associations of each of the early adult life transitions with changes in fast food intake between pairs of waves, following Curran et al. [29], as shown in a path diagram in Fig. 1. Each of these latent factors (int2, int3 and int4) represents an additional intercept which is added to the growth curve at wave 2, 3 and 4 respectively. These additional intercepts are regressed onto the transition variables (T1-T5) and their variance constrained to 0 such that the regression coefficients produced represent a step increase or decrease in fast food intake associated with each transition. For each transition we compare those who went through the transition with a reference category of those whose status did not change between consecutive waves: they did not transition, or stayed in the transitioned state between 2 waves. Since the additional intercept between waves is regressed on all transition variables, the associations of each of these transitions are mutually adjusted.

To examine the associations between fast food intake and life transitions, we developed two separate growth curve models: in the first model, ‘Transitions across all waves’, the association between the transition and the change in fast food intake was constrained to be the same across all waves, providing an overall indication of change in fast food intake associated with that transition, regardless of the timing of the transition during early adulthood. We then removed this constraint, to assess ‘Transitions between pairs of waves’, allowing the association between the transition and fast food intake to vary over time, to look at the effect of a transition occurring between each contiguous pair of waves.

Since adolescents were recruited through schools we also tested whether school-level intraclass correlation coefficients (ICCs) might suggest use of multilevel analysis to adjust for clustering by school. However, ICCs were low (ICC of 0·02 for baseline fast food intake and lower at subsequent waves), which, with an average cluster size of 94, suggests little impact of clustering on these analyses. Therefore, adjustment for school-level clustering was not included.

All models were estimated in Mplus (version 8.3) using the maximum likelihood estimator with robust standard errors (MLR), which is robust to non-normality in the dependent variable. Missing data were addressed using the full information maximum likelihood estimation, under the Missing at Random assumption, with variables related to attrition included as time-invariant covariates in the model. Model fit was assessed using a panel of fit indices: Akaike Information Criterion (AIC), Bayesian Information Criterion (BIC), Root Mean Square Error Of Approximation (RMSEA), Comparative Fit Index (CFI), Standardized Root Mean Square Residual (SRMR).

## Results

### Sample characteristics

Longitudinal data were available from participants from up to four waves of data collection, at mean ages 14.9 (SD = 1.6), 19.4 (SD = 1.7), 25.3 (SD = 1.6) and 31.1 (SD = 1.6) years. Participants (*n* = 2902) who provided data at two or more waves were included in the analysis, since two waves of data were required to allow a life event to be recorded between waves. Baseline descriptive data for the included sample are shown in Table 1. The analytical sample consisted of a greater proportion of participants who were female, non-Hispanic White, and were of higher parental SES than the excluded sample.

**Table 1:** Demographic characteristics of the included sample: participants in the Project EAT study with two or more waves of survey data (*n* = 2902)

	Included sample
Age at wave 1, mean (SD)	14.9 (1.6)
Gender: % female (n)	53.8 (1561)
Race/ethnicity: % non-Hispanic white, (n)	59.8 (1719)
Parental SES, %, (n)	
1 (lowest)	16.2 (464)
2	17.1 (491)
3	23.6 (678)
4	26.9 (771)
5 (highest)	16.2 (466)
Health status: % good or excellent, (n)	65.1 (1869)

### Prevalence and timing of life transitions in the study population

Each of the five transitions were common within our population (Table 2), ranging from 87.1% of individuals leaving full-time education to 43.2% becoming a parent across all the waves of data available. Table 2 also reports the proportion of participants who experienced a transition between each pair of waves. For example, the highest proportion of participants (32.4%) left the parental home between waves 1 and 2. The correlation between timing of transitions was low (< 0.3) for most pairs of transitions. The highest correlation being between leaving education and starting full-time work occurred between waves 1 and 2 and showed a phi coefficient of 0.47. Phi coefficients for associations between each pair of transitions are shown in Table S1 (see supplementary information).

**Table 2:** Descriptive data on life transitions among participants in the Project EAT study with two or more waves of survey data (*n* = 2902)

	Across all waves	Between pairs of waves		
		Waves 1–2	Waves 2–3	Waves 3–4
**Leaving the parental home**				
Completes transition, %(n)	76.3 (2214)	32.4 (940)	29.7 (863)	14.2 (411)
No transition, %(n)	22.8 (661)	54.3 (1576)	37.3 (1083)	43.8 (1271)
Missing, %(n)	0.9 (27)	13.3 (386)	32.9 (956)	42.0 (1220)
**Leaving full-time education**				
Completes transition, %(n)	87.1 (2527)	26.3 (763)	49.0 (1423)	11.8 (341)
No transition, %(n)	12.3 (357)	58.6 (1700)	27.2 (789)	47.9 (1390)
Missing, %(n)	0.6 (18)	15.1 (439)	23.8 (690)	40.4 (1171)
**Beginning full-time employment**				
Completes transition, %(n)	74.7 (2169)	26.5 (769)	35.8 (1038)	12.5 (362)
No transition, %(n)	24.0 (697)	58.6 (1701)	34.8 (1010)	41.7 (1210)
Missing, %(n)	1.2 (36)	14.9 (432)	29.4 (854)	45.8 (1330)
**Beginning cohabitation**				
Completes transition, %(n)	59.9 (1739)	10.5 (305)	31.9 (925)	17.5 (509)
No transition, %(n)	39.5 (1145)	76.2 (2211)	39.8 (1155)	40.3 (1169)
Missing, %(n)	0.6 (18)	13.3 (386)	28.3 (822)	42.2 (1224)
**Becoming a parent**				
Completes transition, %(n)	43.2 (1254)	9.7 (281)	15.6 (454)	17.9 (519)
No transition, %(n)	56.1 (1627)	75.7 (2197)	61.5 (1784)	41.5 (1205)
Missing, %(n)	0.7 (21)	14.6 (424)	22.9 (664)	40.6 (1178)

‘No transition’ includes both those who have not yet transitioned or who have already completed a transition, i.e. those who show no change in status between 2 waves. Mean age at each wave: wave 1: 14.9y (SD = 1.6), wave 2: 19.4y (SD = 1.7), wave 3: 25.3y (SD = 1.6) and wave 4: 31.1y (SD = 1.6)

### Trajectory of fast food intake with age

**Fig. 2:**
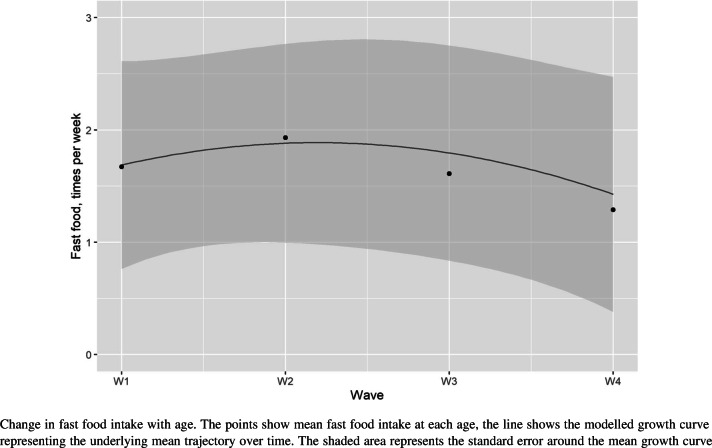
Change in fast food intake with age

We developed a growth model to capture changes in fast food intake with age and across transitions, across all four waves of data. We first tested model fit of the unconditional growth model with either a linear, quadratic or a non-linear model with estimated time scores, finding that model fit was best with an underlying quadratic model (see Table S2, supplementary information). Model fit further improved with the addition of covariates and additional latent intercepts associated with between-wave transitions. The full model (Fig. 1) showed good model fit with RMSEA = 0.015, CFI = 0.957, SRMR = 0.011. Based on this full model, we report an underlying growth curve with intercept 1.69 (SE 0.03), linear slope 0.33 (SE 0.06), and quadratic slope − 0.14 (SE 0.02). Figure 2 shows the modelled trajectory of fast food intake over time, together with mean reported fast food intake at each age. This growth curve represents the mean trajectory of a population who do not go through any of the transitions. This trajectory shows that fast food intake increased through adolescence to a maximum of 1.88 (SE 0.94) times/week and then decreased again through early adulthood to 0.76 (SE 2.06) times/week at wave 4 (Fig. 2). Associations between the growth trajectory and time-invariant covariates are shown in Table S3.

### Associations between life transitions and changes in fast food intake

Associations with changes in fast food intake were observed for all life transitions except for leaving full-time education. Leaving the parental home and beginning cohabitation were both associated with decreases in fast food intake while beginning full-time employment and having a child were associated with increases in fast food intake (Table 3, model 1). When considered by wave, a number of transitions only show statistically significant associations with fast food intake between specific waves (Table 3, model 2). For example, the decrease in fast food intake associated with leaving the parental home reduced with age, while the decrease in fast food intake on beginning cohabitation increased with age. These time-specific effects should be interpreted with respect to the underlying growth curve (Fig. 2), and other ongoing transitions. For example, a decrease in fast food intake of − 0.25 times per week on leaving the parental home between waves 1 and 2 is set against the underlying upward trend at this time, suggesting that, on average, leaving the parental home was associated with flattening the underlying increase in fast food seen over this period, rather than, on average, a decrease in intake.

**Table 3:** Association of transtions with additional change in fast food intake, beyond the underlying trajectory, among participants in the Project EAT study with two or more waves of survey data (*n* = 2902)

	Change in fast food intake, times per week, across transitions, β (*p*-value)			
	Model 1: Transitions across all waves	Model 2: Transitions between pairs of waves		
Transition	All waves	Waves 1–2	Waves 2–3	Waves 3–4
Leaving the parental home	−0.17 (0.004)	−0.25 (< 0.001)	− 0.15 (0.03)	0.01 (0.91)
Leaving full-time education	−0.01 (0.90)	0.04 (0.64)	−0.05 (0.48)	− 0.07 (0.54)
Beginning full-time employment	0.16 (0.004)	0.22 (0.001)	0.11 (0.10)	0.20 (0.06)
Beginning cohabitation	−0.16 (0.007)	−0.05 (0.60)	− 0.17 (0.01)	−0.20 (0.02)
Becoming a parent	0.16 (0.004)	0.19 (0.05)	0.15 (0.05)	0.13 (0.08)

Model 1 constrains associations to be the same across all waves, while Model 2 allows associations to vary over time. In other respects these models are identical. Models are adjusted for time-invariant covariates, the underlying growth curve, and mutually adjusted for the other transitions (see Fig. 1). Mean age at each wave: wave 1: 14.9y (SD = 1.6), wave 2: 19.4y (SD = 1.7), wave 3: 25.3y (SD = 1.6) and wave 4: 31.1y (SD = 1.6)

## Discussion

In this longitudinal analysis of young adults, we explored how fast food intake changed through early adulthood, and how five life transitions of early adulthood are associated with changes in fast food intake. Factors contributing to increases in fast food intake were beginning full-time employment and becoming a parent. The strongest association between these transitions and fast food intake was seen between waves 1 and 2 (mean age 15–19 years), when the effect of these transitions was in addition to an underlying increase of a similar magnitude over this period. Leaving the parental home and beginning cohabitation were associated with decreases in fast food intake, while leaving full-time education was not associated with a change in fast food consumption. These findings suggests that those starting employment and starting a family early in life are at the highest risk for increases in fast food intake, and public health policy or interventions may benefit from focus on these groups.

### Comparison with previous research

The frequency of fast food intake seen in this cohort is slightly lower than that reported in comparable U.S. population datasets. Data from the National Longitudinal Study of Adolescent Health (Add Health), collected at around the same time as the Project EAT study (1996–2002), and using a very similar measure, reported a fast food consumption frequency of 2.15 (SE 0.05) times per week at age 16, and 2.48 (SE 0.05) times per week at age 21 [13]. Meanwhile, recent data from the U.S. National Health and Nutrition Examination Survey (2013–2016), collected using a 24-h dietary recall, reported that 45% of young adults (aged 20–39) reported consuming fast food on a given day [9], with an overall decrease in fast food consumption with age among U.S adults [9].

There are very limited data on changes in diet and eating behaviours across early adulthood life transitions, as reported in our recent systematic reviews [20, 21], and we have found no other studies that systematically looked at changes in fast food intake across life transitions. Our finding of a relative decrease in fast food intake on leaving the parental home may initially seem counterintuitive. Previous research has suggested that consumption of meals with the family is associated with higher diet quality in adolescence [30], and we have previously shown that leaving the parental home is associated with a decrease in fruit and vegetable consumption in early adulthood in a Norwegian cohort [18]. One explanation for the decrease in fast food intake seen in the current study on leaving the parental home could be a decrease in financial resources in this group. Studies of U.S. adolescents and young adults has shown high price sensitivity to fast food, with an increase in price associated with a decrease in frequency of fast food consumption [31, 32]. Young adults may therefore reduce, or limit, their increase in fast food intake when they are no longer supported financially by their family. This explanation would fit with our finding that this decrease is seen primarily at younger ages, and the association reduces in later waves of this study. Another factor to consider is the environment to which young people are moving. A portion of our participants will be moving to live on college and university campuses, where institutional meal provision arrangements may reduce the need for further food purchase from fast food restaurants. Previous research in Project EAT has shown that young women living on campus consumed fast food less frequently than those living with parents or in rented accommodation, after adjusting for sociodemographic covariates [33]. Others have further suggested that those who live with their families may use fast food restaurants as a social space to spend time with friends outside the parental home [34, 35], so this need may diminish following a move to living independently.

We did not see any changes in fast food intake on the transition out of education. A previous study reported increases in confectionery and sugar-sweetened beverage consumption over this transition [18], but the mechanisms may be different for fast food intake. We found that starting full-time employment was associated with an increase in fast food intake. This would fit with an economic explanation of changes in fast food purchasing, since on beginning employment participants would be expected to have greater financial resources. Another contributing factor could be a decrease in time available for food preparation as a result of an increase in time spent working and commuting when entering full-time employment.

Qualitative studies have suggested that many changes in eating behaviours and diet can occur due to new partnerships, where those newly cohabiting/married typically negotiate and compromise to allow them to share food purchasing and preparation and enjoy meals together [36, 37]. Our finding of a decrease in fast food intake as individuals start cohabitation may reflect a change to more home cooking with a partner. Married participants of the Coronary Artery Risk Development in Young Adults (CARDIA) study were reported to live in neighbourhoods with lower poverty and lower fast food restaurant density [38], therefore the change in consumption may also reflect a change in physical living environments and fast food availability. Similar to the findings reported here, an Australian longitudinal study reported a greater decrease in fat and saturated fat consumption from age 18 to 25 years among men (but not women) living with a partner at 25 years, compared to those without a partner [39].

Finally, we found that fast food intake increased among those who became a parent for the first time. This is consistent with the use of fast food for time-saving and convenience [34]. This finding is also consistent with analysis of the Australian Longitudinal Study on Women’s Health, where starting a family was associated with an increase in consumption of high-fat and sugar diets, as well as an increase in total energy intake [40]. Data from the CARDIA study reported no difference in change in fast food intake over a 7 year period between those who became parents and non-parents [41]. However, the CARDIA cohort were older than the Project EAT cohort, aged 25y at baseline, and this observation is therefore in agreement with the decreasing strength of association across later waves that we find in these analyses.

### Strengths and limitations of the study

This is the first analysis, to our knowledge, to investigate longitudinal associations between multiple life transitions and changes in fast food intake across early adulthood. Strengths of this analysis include the use of data from the longitudinal Project EAT cohort, which allowed us to study changes in fast food intake over a 15 year period including early adulthood, a notoriously difficult period of life to study [42]. Data on a wide range of social measures, including living arrangements, educational and employment status, and children, allowed us to assess associations between fast food intake and individual transitions independently, controlling for co-occurrence of other transitions.

One limitation of this dataset was the simplicity of the fast food measure. Intake of fast food was based on only a single question on frequency of consumption, with a categorical response option, which although it is similar to measures used elsewhere, has not been validated against other dietary assessment methods [13]. In addition, respondents were asked to report on consumption during the ‘last week’ which may not have been a typical week for all participants. These limitations may lead to greater measurement error than would be expected from a more detailed questionnaire. However, a strength is that the same question was repeated across all waves, so it is likely that any response bias would be consistent across waves and would not affect our analysis of changes in intake. We have previously reported secular trends in adolescent fast food intake in the Minneapolis/St. Paul population, which suggest an overall decrease in fast food intake over a similar time frame to this study [43]. These secular trends may have affected the shape of our underlying fast food trajectory, as study participants will have been responding to macrolevel societal and environmental changes as well as individual developmental changes with age. However, we would not expect these secular trends to affect the associations seen between life transitions and fast food intake since our model accounts for the underlying fast food trajectory. A further limitation is that data were collected at only four time points, which were each 5 years apart. As such we were not able to assess the immediate impact of a transition, but only the longer-term impact of a transition which may have occurred at any time over the last 5 years. Because of this limitation, and the low number of data points available, we did not assess whether a transition resulted in a change in the slope of fast food intake, only whether a step change in fast food intake was seen between one wave and the next. As with all observational cohort studies there is a trade-off between a desire for detailed data collection on one aspect of the study, interest in a wide range of social and behavioural measures, and the level of data collection burden that participants can be expected to tolerate. Nevertheless, the ability of this data to show associations between transitions and changes in intake, despite the expected noise in the outcome variable, indicates the strength of these findings. Further research on changes in more detailed measures of diet across these transitions will be able to confirm or refute these findings. Given that this cohort were recruited from a metropolitan area of Minnesota, it would also be of interest to investigate in future analyses whether the impact of these life transitions is the same in other populations, as well as differences between different population groups, for example whether associations differ by gender or socioeconomic status.

## Conclusions

Our analyses find that two of the five life transitions of early adulthood, beginning full-time employment and becoming a parent, are associated with increases in fast food intake. The strongest associations were seen between waves 1 and 2 (mean age 15 to 19 years), when each of these transitions was associated with an average increase in fast food intake of around 0.2 times per week, in addition to the underlying age-related increase of a similar magnitude over this period. Our findings suggest that public health policy or interventions designed to reduce fast food intake in young adults may benefit from particular focus on those beginning full-time employment and becoming a parent, to ameliorate the impact of these transitions. Development of interventions in collaboration with employers recruiting young workers or parent education programs for young parents may facilitate delivery of interventions to the high risk groups identified in this study.

## Supplementary information

**Additional file 1 : Table S1.** Phi coefficients for associations between pairs of transitions. **Table S2.** Model fit statistics for tested growth models. **Table S3.** Association of time-invariant covariates with latent growth factors in the final model.

## Data Availability

The datasets generated and/or analysed during the current study are not publicly available but are available from the senior author (DNS) on reasonable request.

## Abbreviations

Add Health: National Longitudinal Study of Adolescent Health
AIC: Akaike information criterion
BIC: Bayesian Information Criterion
CARDIA: Coronary artery risk development in young adults study
CFI: Comparative fit index
ICC: Intraclass correlation coefficient
MLR: Maximum likelihood estimator with robust standard errors
Project EAT: Project eating and activity in teens and young adults
RMSEA: Root mean square error of approximation
SE: Standard error
SES: Socioeconomic status
SRMR: Standardized root mean square residual
U.S.: The United States of America
