# Supplementary material for: Associations of early adulthood life transitions with changes in fast food intake: a latent trajectory analysis
Source: Int J Behav Nutr Phys Act. 2020 Oct 9;17:130. doi: 10.1186/s12966-020-01024-4 (PMC7547405; doi:10.1186/s12966-020-01024-4)
Supplement: Supplementary file 1 — Additional file 1 : Table S1. Phi coefficients for associations between pairs of transitions. Table S2. Model fit statistics for tested growth models. Table S3. Association of time-invariant covariates with latent growth factors in the final model. [file 12966_2020_1024_MOESM1_ESM.docx]

# Supplementary Tables

Table S1: Phi coefficients for associations between pairs of transitions

|  | Phi coefficients of associations between pairs of transitions | | | | | | | | |
| --- | --- | --- | --- | --- | --- | --- | --- | --- | --- |
|  | Leaving family home | Beginning cohabitation | | Leaving education | | Beginning employment | | Having a child | |
| Wave 1-2 | | | | | | | | | |
| Leaving the family home | 1.00 | |  | |  | |  | |  |
| Beginning cohabitation | 0.27 | | 1.00 | |  | |  | |  |
| Leaving full-time education | 0.09 | | 0.34 | | 1.00 | |  | |  |
| Beginning full-time employment | 0.23 | | 0.20 | | 0.46 | | 1.00 | |  |
| Having a child | 0.04 | | 0.32 | | 0.26 | | 0.03 | | 1.00 |
| Wave 2-3 | | | | | | | | | |
| Leaving the family home | 1.00 | |  | |  | |  | |  |
| Beginning cohabitation | 0.20 | | 1.00 | |  | |  | |  |
| Leaving full-time education | -0.01 | | 0.13 | | 1.00 | |  | |  |
| Beginning full-time employment | 0.07 | | 0.04 | | 0.30 | | 1.00 | |  |
| Having a child | 0.09 | | 0.24 | | 0.00 | | -0.02 | | 1.00 |
| Wave 3-4 | | | | | | | | | |
| Leaving the family home | 1.00 | |  | |  | |  | |  |
| Beginning cohabitation | 0.18 | | 1.00 | |  | |  | |  |
| Leaving full-time education | 0.18 | | 0.18 | | 1.00 | |  | |  |
| Beginning full-time employment | 0.14 | | 0.13 | | 0.37 | | 1.00 | |  |
| Having a child | -0.06 | | 0.12 | | -0.01 | | -0.06 | | 1.00 |

Table S2: Model Fit statistics for tested growth models

| **Fit index** | **Unconditional linear growth model** | **Unconditional quadratic growth model** | **Full model with covariates and all transitions** |
| --- | --- | --- | --- |
| AIC | 36108.413 | 36015.301 | - |
| BIC | 36162.168 | 36092.948 | - |
| RMSEA | 0.082 | 0.080 | 0.015 |
| CFI | 0.736 | 0.951 | 0.957 |
| SRMR | 0.054 | 0.022 | 0.011 |

Table S3: Association of time-invariant covariates with latent growth factors in the final model

|  | Fast food intake growth parameters (units: times fast food consumed per week) | | |
| --- | --- | --- | --- |
|  | Intercept,  *β* (p-value) | Linear slope  *β* (p-value) | Quadratic slope  *β* (p-value) |
| Age (yrs) | 0.06 (0.002) | -0.12 (<0.001) | 0.03 (0.002) |
| Gender  (female vs male) | -0.15 (0.01) | -0.52 (<0.001) | 0.14 (<0.001) |
| Race/ethnicity (non-white vs white) | 0.20 (0.004) | -0.15 (0.20) | 0.05 (0.16) |
| Parental SES  (1= low SES, 5= high ses) | -0.06 (0.01) | -0.04 (0.38) | 0.01 (0.56) |
| Baseline health status (1=poor, 4=excellent) | -0.11 (0.08) | -0.15 (0.12) | 0.04 (0.16) |

Values indicate change in fast food intake growth parameters for one unit or category change in each covariate.
